# The measurement of stroke volume by cine magnetic resonance imaging and phase contrast velocity mapping

**DOI:** 10.1186/1532-429X-15-S1-E105

**Published:** 2013-01-30

**Authors:** Takashi Tanimoto, Shingo Ota, Kohei Ishibashi, Takashi Yamano, Yasushi Ino, Tomoyuki Yamaguchi, Kumiko Hirata, Takashi Kubo, Imanishi Toshio, Takashi Akasaka

**Affiliations:** 1 Cardiovascular Medicine, Wakayama Medical University, Wakayama city, Japan

## Background

In the assessment of left ventricular stroke volume in cine magnetic resonance, drawing the endocardial contours in short axis slices are difficult at the 1 or 2 most basal slices due to systolic movement of the base towards the apex. The forward flow in the ascending aorta can be quantified using phase contrast velocity mapping. This study investigated which basal slices should be included in the assessment of left ventricular stroke volume, compared with those of phase contrast velocity mapping.

## Methods

In 46 patients with various type of heart disease, multi slice breath-hold cine imaging of short axis views covering the entire left ventricle were obtained using 1.5 Tesla clinical scanner with 32-channel coil. The cases with mitral regurgitation were excluded. The endocardial contours in short axis views were drawn semi-automatically from left ventricular apex to 3 different basal slices, trace A; circular left ventricular endocardial contours in both end-diastole and end-systole, trace B; uninterrupted endocardial contour in end-diastole and interrupted endocardial contour by left ventricular outflow tract in end-systole, and trace C; interrupted endocardial contours with left ventricular outflow tract even in end-diastole. The most basal segment with muscle less than half of the circumference was excluded for analysis. The measured stroke volumes were compared with those of obtained by phase contrast velocity mapping in the ascending aorta.

## Results

The total short axis slices included in the analysis were 6.7±1.6, 7.7±1.6, and 8.7±1.6 in trace A, B, and C, respectively. The stroke volume by cine imaging were 49±13 ml, 58±14 ml, and 65±15 ml in trace A, B, and C, respectively. The forward flow in the ascending aorta was 66±18 ml by phase contrast mapping, which showed good agreement only with trace C (p<0.001).

## Conclusions

The basal short axis slice with interrupted endocardial contour by outflow tract should be included for tracing in the assessment of left ventricular chamber size and ejection fraction.

## Funding

None.

**Figure 1:**
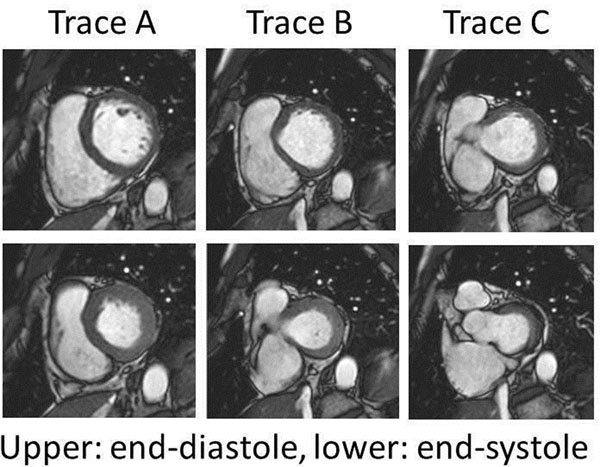
Representative basal short axis slices.

